# Nanoindentation of Aluminum Single Crystals: Experimental Study on Influencing Factors

**DOI:** 10.3390/ma12223688

**Published:** 2019-11-08

**Authors:** Pavel Filippov, Ursula Koch

**Affiliations:** 1 Department of Earth- and Environmental Science, Ludwig-Maximilian-University, 80333 Munich, Germany; 2 Munich University of Applied Sciences, 80335 Munich, Germany; u.koch@hm.edu

**Keywords:** nanoindentation, aluminum, single crystal, micromechanics

## Abstract

Results from nanoindentation of aluminum single crystals deliver valuable information as model systems for understanding technical aluminum alloys. The effect of the crystal orientation and the azimuthal indenter orientation on indentation hardness and modulus was studied by Vickers indentation (max. load 10 mN) on single crystal surfaces with (100), (110), and (111) orientations. The average indentation hardness varied, depending on the crystallographic orientation, by 1.8%. The anisotropy of the elastic modulus (1.1% of the average modulus) is lowered (indentation averaging effect). This is predicted by explicit approximation of the contact problem (conical indenter, orthotropic material). It was found that indentation hardness and modulus vary periodically with the azimuthal indenter orientation on (100)- and (110)-oriented surfaces (relative amplitude of 1.8% for indentation hardness and 2.6% of the modulus). This is attributed to the combined effect of the indenter geometry and crystal symmetry. For the first time, this effect was quantified for aluminum single crystals.

## 1. Introduction

Nanoindentation is a well-developed and widely used method for determining hardness and elastic modulus on different length scales ranging from several hundred micrometers to a few nanometers [1,2]. This method is widely used to access nanomechanical properties of crystalline and amorphous and heterogeneous materials such as glasses, ceramics, or metals [3,4,5]. These properties are essential for micro-manufacturing of metallic materials. However, the results for the elastic modulus of metals achieved by nanoindentation often differ from theoretical values, as well as from results obtained from macroscopic methods (i.e., tensile or ultrasonic test), as pointed out by Shuman et al. [6].

Different approaches have been used to improve the nanoindentation results for metals. For example, special indentation procedures have been developed by Shuman et al. [6] to deal with the overestimated elastic modulus of different materials. Nemecek [7] has developed a method to analyze heterogeneous structure materials and aluminum foams. Furthermore, attempts have been made to achieve a better understanding of the indentation process through simulation [8,9,10].

In order to understand the indentation response of materials such as polycrystalline metals and alloys, it is essential to have a solid understanding of the base material. In order to shed more light on the indentation of aluminum materials, we have chosen aluminum single crystals of three basic orientations as a model system.

The influence of the crystallographic orientation has been studied for different materials. For example, it has been investigated on the basis of polycrystalline material for austenitic steel by Hausild et al. [11] and for titanium by Merson et al. [12]. Among the face-centered cubic (fcc) metals, copper has been studied in many aspects. For example, Zahedi et al. [13] found the crystal orientation to have only a small effect on the indentation results; Geng et al. [14] has analyzed the influence of crystallographic orientation with respect to nanoscratching behavior and Liu et al. [15] has investigated the plastic behavior of single crystal copper by numerical simulation and indentation with a spherical indenter. Although copper and aluminum both possess the fcc crystal system, it is impossible to transfer results because of their different anisotropic ratios.

Few dedicated experimental studies on the indentation of aluminum single crystals have been published. In their basic and important study, Vlassak and Nix [16] investigated the directional dependence of elastic properties of aluminum single crystals among other materials. Yet, their work focuses on mathematical modeling of the contact problem and the experimental results have no statistical basis. In a recent study by Liu et al. [8], they combine simulational and experimental approaches. Here, indentation results of Al single crystals are included but without statistical analysis. Moreover, the absolute values and anisotropy found by Liu differ significantly from the results reported by Vlassak and Nix [16]. There are other studies on the indentation of aluminum single crystals, i.e., by Koloor et al. [17], but they often only demonstrate the indentation results of one crystallographic orientation or are lacking statistical analysis. Statistical results of all three crystallographic orientations are desired for a better understanding of the material.

The aim of this research is to create an experimental basis for the dependency of indentation hardness and modulus on the crystallographic orientation of aluminum. This includes statistical analysis of the indentation results (hardness and modulus). A comparison of the indentation results to literature and approximations of the indentation contact problem (described in Section 2.2) are done to verify the experimental results. To achieve the experimental data, grid indentation was performed on carefully prepared high-purity aluminum single crystals in three major crystallographic orientations (100), (110), (111).

To complement dependency on the crystallographic orientation, the influences of the azimuthal orientation of the indenter on the sample surface have been investigated. According to Wang et al. [18], there is a coupled effect of indenter geometry and crystallographic orientation. This effect is studied quantitatively in this work.

## 2. Theoretical Background

### 2.1. Young’s Modulus

Young’s modulus E possesses a significant anisotropy for certain materials (anisotropy factor 1.23 for aluminum, fcc lattice type). E in the direction [uvw] can be calculated according to the following equation (cubic crystal lattice materials) [19,20,21,22]:

(1) $$\frac{1}{E_{\left[uvw\right]}}=S_{11}-\left[2\left(S_{11}-S_{12}\right)-S_{44}\right]\left(\alpha^{2}\beta^{2}+\alpha^{2}\gamma^{2}+\beta^{2}+\gamma^{2}\right)$$

$E_{\left[uvw\right]}$ is Young’s modulus in an arbitrary spatial direction [uvw] and α, β, γ are the respective direction cosines. The components of the compliance matrix $S_{11}$, $S_{12}$, and $S_{44}$ can be converted to the corresponding components of the elasticity matrix, i.e., the elastic constants $C_{11}$, $C_{12}$, $C_{44}$. These constants are well known; here we used the values also used by Vlassak and Nix [16] according to Simmons and Wang [23] with $C_{11}$ = 107.3 GPa, $C_{12}$ = 60.9 GPa, and $C_{44}$ = 28.3 GPa. By this means directional Young’s Moduli $E_{\left[usw\right]}$ for three major crystallographic directions can be calculated, resulting in $E_{100}$ = 63.2 GPa, $E_{110}$ = 72.0 GPa, and $E_{111}$ = 75.6 GPa.

### 2.2. Indentation Modulus

The indentation modulus M is determined by the indentation measurements. It is the result of averaging Young’s modulus over all directions depending on the crystal symmetry, elastic constants, and the indenter geometry. Therefore, M can differ from the directional Young’s modulus calculated for the indentation direction. Usually, the anisotropy (i.e., the difference between the stiffest and the softest direction) of M is lower than the anisotropy of Young’s modulus. As explained by Göken [24] this is often referred to as the indentation averaging effect.

Vlassak and Nix [25] calculated M of cubic crystals for flat indenter geometries (flat circular punch and a paraboloid). They then extended the solution of the contact problem to a flat triangular indenter. Indentation moduli for some cubic materials including aluminum are given in [16]. Since the analytical solution to the proposed equations was not available, they were solved numerically. The results indicate a decrease in the measured anisotropy and verify the indentation averaging effect.

Additionally, there is an analytic approach for the calculation of the indentation moduli of cubic materials. The solution of the contact problem can be approximated by assuming the indenter to be conical (rotational symmetry) and the material to be orthotropic. This explicit (analytic) solution of the contact problem is proposed by Delafargue and Ulm [26]. It is shown by Delobelle et al. [27] to be in good agreement with the calculation method proposed by Vlassak and Nix [16].

In this work we utilize the formulation used by Delobelle et al. [27], which give very similar results to the three-dimensional finite element simulation of Berkovich indentation. Thus, the indentation modulus $M_{\langle uvw\rangle }$ in the arbitrary crystallographic direction 〈uvw〉 can be calculated as follows:

(2) $$M_{\langle uvw\rangle }=M_{VRH}\sqrt{\frac{\langle C_{11}^{*}\rangle }{\langle C_{33}^{*}\rangle }}$$

with $M_{VRH}$ being the isotropic indentation modulus approximated with the Voigt–Reuss–Hill method and $\langle C_{ij}^{*}\rangle$ the values of the elastic constants in the indentation direction.

$M_{VRH}$ can be calculated from the isotropic shear moduli $G_{V}$, $G_{R}$, and $G_{VRH}$ (Voigt, Reuss, and Hill approximations respectively, i.e., given by Chung and Buessem [28]):

(3) $$\begin{array}{c} G_{V}=\frac{C_{11}-C_{12}+3C_{44}}{5}\\ G_{R}=\frac{5}{\frac{4}{C_{11}-C_{12}}+\frac{3}{C_{44}}}\\ G_{VRH}=\frac{G_{V}+G_{R}}{2} \end{array}$$

The isotropic Young’s modulus $E_{VRH}$ can be calculated from $G_{VRH}$:

(4) $$E_{VHR}=G_{VRH}\left(2+2\nu \right)$$

Finally, $M_{VRH}$ can be achieved according to according to the relation used by Göken [24]:

(5) $$M_{uvw}=E_{uvw}/\left(1-\nu^{2}\right)$$

The aluminum Poisson’s ratio ν used for the calculations is 0.35 [21]. This relation is also used compare Young’s modulus from Equation (1) to the indentation modulus.

The elastic constants in the indentation direction can be calculated using the elastic constants used by Vlassak and Nix [16,23] and the following relations for $\langle C_{ij}^{*}\rangle$ according to Delobelle et al. [27]:

(6) $$\begin{array}{ccccc} \langle 100\rangle & & \langle C_{11}^{*}\rangle =C_{11} & & \langle C_{22}^{*}\rangle =\frac{3C_{11}+C_{12}+2C_{44}}{4}\\ \langle 110\rangle & & \langle C_{11}^{*}\rangle =\frac{C_{11}+C_{12}+2C_{44}}{2} & & \langle C_{22}^{*}\rangle =\frac{9C_{11}+7C_{12}+14C_{44}}{16}\\ 111 & & C_{11}^{*}=\frac{C_{11}+2C_{12}+4C_{44}}{3} & & C_{22}^{*}=\frac{C_{11}+C_{12}+2C_{44}}{2} \end{array}$$

The anisotropy of indentation hardness depends on a more complicated plastic material behavior which is difficult to derive explicitly from crystal properties.

### 2.3. Determination of the Indentation Hardness and Modulus from Indentation Curves

Indentation hardness $H_{IT}$ and indentation modulus M are determined according to the Oliver and Pharr method [2], which was based on the original work of Doerner and Nix [1] (implemented in the normative [29]). Hence, the $H_{IT}$ is defined as follows:

(7) $$H_{IT}=\frac{F_{max}}{A_{p}\left(h_{c}\right)}$$

with the maximum load $F_{max}$ and the projected contact area $A_{P}\left(h_{c}\right)$ of the indenter ($A_{p}=24.5\cdot h_{c}$ for a Vickers indenter). M is defined according to:

(8) $$M={\left(\frac{1}{\frac{\sqrt{\pi }\cdot S}{2\sqrt{A_{p}\left(h_{c}\right)}}}-\frac{{\left(1-\nu_{i}\right)}^{2}}{E_{i}}\right)}^{-1}$$

Here, $\nu_{i}$ is the Poisson’s ratio of the sample and of the indenter respectively. $E_{i}$ is the Young’s modulus of the indenter. The contact stiffness S is determined by the numerical fit of a power law to the unload-part of the load–displacement curve. The used $E_{i}$ and $\nu_{i}$ of the diamond-indenter are 1140 GPa and 0.07 respectively [29,30].

### 2.4. Statistical Pop-In Analysis

In order to allow sufficient plastic deformation of materials with low dislocation density, the activation energy necessary to generate and move dislocations must be exceeded. This takes place at the low load phase of the indentation process. This is noticeable as a discontinuity (pop-in) in the nanoindentation of metal single crystals naturally possessing low dislocation density. The presence of the pop-in indicates that the metal is locally dislocation free. The load–displacement curves of strain hardened metals show a continuous increase of the depth with applied load (no pop-in effect) [31,32]. As has been demonstrated by Wang [33], a statistical pop-in analysis can be used to estimate the surface preparation quality.

## 3. Materials and Methods

### 3.1. Materials and Sample Preparation

This study used commercially available, high purity (99.999%) aluminum single crystal grown in the <111> direction. Firstly, the orientation of the obtained single crystal (cylinder, diameter 10 mm and length 25 mm) was checked with the X-Ray Laue (Crystallography Section of the Department of Earth- and Environmental Science, Ludwig-Maximilian-University, 80333 Munich, Germany, Prof. Dr. P. Gille) diffraction method. Subsequently, three sections along (111), (110), and (100) lattice planes were cut by a gentle lapping saw.

These samples were embedded in commercially available cold-mounting resin. In order to avoid strain hardening of the single crystal surface, a special combination of mechanical grinding and electrolytic polishing was used. After grinding (2500 SiC-paper), the samples were polished electrolytically in a percloric acid-based electrolyte “CT A2” (Cloeren Technology GmbH, Wegberg, Germany) for 60 sec at 30 V and subsequently cleaned with distilled water and ethanol. After polishing the backside of the specimen, the specimen was ground plane-parallel to the front side, with the maximum angle between the front- and back-side of 0.3°.

### 3.2. Pile-Up and Sink-In Corrections

The inevitability of plastic behavior (i.e., pile-up of sink-in) correction was considered on the basis of two different correction methods. According to the work of McElhaney et al. [34], the correction factor $\alpha =A_{p}/A_{c}$ (with a projected area $A_{p}$ and the cross-sectional area of the indenter $A_{c}$) is determined on the basis of scanning electron microscope (SEM) images. To achieve that, SEM images of three exemplary indents for each crystallographic orientation and indenter–sample orientation were captured. $A_{p}$ was determined manually based on the indenter contour, while $A_{c}$ was calculated from the indent diagonals. Alternatively, the hardness $H_{p}$ was calculated for a representative indentation curve on the basis of plastic and elastic indentation work. This method is proposed by Tuck et al. [35]:

(9) $$H_{p}=kF_{max}^{3}/9W_{p}^{2}$$

$W_{p}$ is the plastic work of indentation which is determined as the area enclosed by the load–depth curve. k is a constant typical for each indenter geometry (for Vicker’s indenter k=0.0378 [35]).

### 3.3. Indentation Setup

Nanoindentation experiments were performed with the “Picodentor HM500” (Fischer Technology Inc., Windsor, CT, USA) equipped with a Vickers (London, UK) diamond indenter (tip radius approx. 480 nm). The load resolution of the Picodentor HM500 is higher than 100 nN (generated via an electromagnetic induction coil) and the resolution of the indentation depth is higher than 40 pm. The instrument is mounted on an active piezoelectric table and placed in a sound isolation housing to minimize acoustic and ground vibrations.

A single set of parameters was used for all indentation experiments. The used $F_{max}$ was 10 mN, while the load and unload times were both 10 s. The square root of the load over time was held constant ($d\sqrt{F}/dt=constant$) during the load and unload phase. This allows for a very gentle low load phase of the indentation, which is extraordinarily important for ductile aluminum single crystals.

To investigate the influence of the crystal orientation, 6 × 6 indentation arrays were executed on samples having (100), (110), and (111) crystallographic orientations. To assert the dependency of the derived mechanical properties on azimuthal indenter orientation, the samples were rotated on a specially designed rotational sample holder from 5° to 180° with 5° steps, while three indentations were made for each angle modulation. The rotation angle of the sample was set randomly to avoid systematic errors.

The distance between the indents was set to approximately ten times the indent diameter to avoid the mutual influence of single indents (twice as much as the normative demands [29]). The indentation experiments were performed in the middle of the specimens to avoid edge effects. All experiments were carried out at room temperature.

### 3.4. Design of the Fit Function

Preliminary experiments have shown a periodic dependency of $H_{IT}$ and M from azimuthal indenter orientation on the sample surface. As shown by Legendre and Duttileul [36], sinusoidal functions are widely used to model periodic phenomena. Therefore, a sine function based on an undamped harmonic oscillation (i.e., Meschede et al. [37]) was used to quantify the influence of the indenter orientation:

(10) $$y\left(x\right)=A+y_{0}\sin\left(2\pi \frac{1}{T}x+\phi_{0}\right)$$

Here, y(x) represents either $H_{IT}$ or M at the given angle x. The angle x depicts the rotation of the sample set on the device. The y-axis-offset A represents the average value $H_{IT}$ or M. The amplitude $y_{0}$ is correlated to the magnitude of the combined effect of the crystal orientation and indenter geometry. The phase angle $\phi_{0}$ with the period duration T describe the azimuthal indenter orientation on the sample surface with regard to the set angle x.

Since the Vicker’s pyramid has four sides, T was fixed at 90°, while other function parameters parameters were fitted with the scaled Lavenberg–Marquardt algorithm by SciDAVis software (version 1.2). Coefficient of determination $R^{2}$ (fraction of the variance unexpailned by the fit function) and standard deviation σ were determined for each data set. Additionally, the fitted $y_{o}$ is presented in the results.

## 4. Results

### 4.1. Quality of Sample Preparation

**Figure 1:**
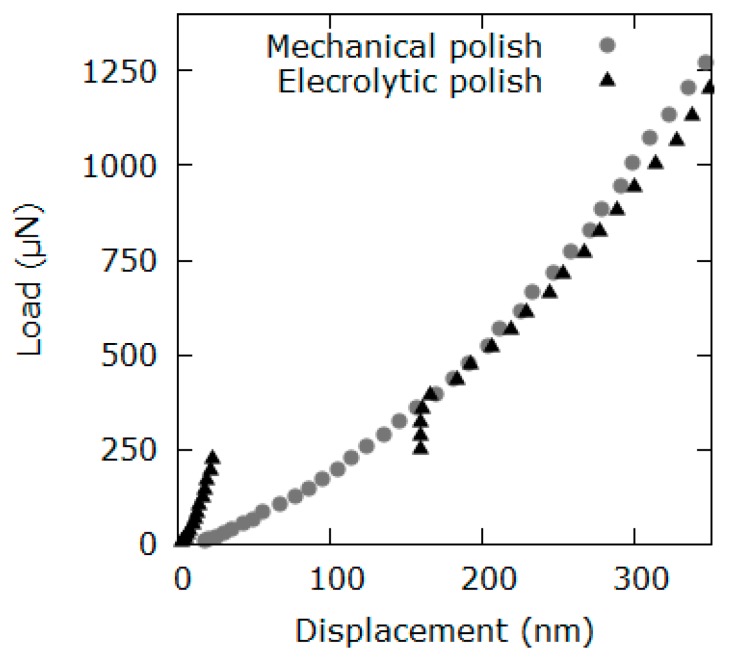
Load–displacement curve on the aluminum (111)-single-crystal surface, polished electrolytically (pop-in) and mechanically (no pop-in).

To verify the quality of the surface preparation (strain hardening), the pop-in effect was analyzed quantitatively. The effect of surface preparation on the pop-in behavior is shown in Figure 1. Two exemplary indentation curves of aluminum (111) single crystal, polished electrolytically and mechanically, are shown here. The curve with the pop-in (electrolytic polish) can be clearly distinguished from the curve without the pop-in. Also, pop-ins are observable in the actual indentation curves shown in Figure 5. The proportion of the load–depth curves showing pop-ins to the total number of curves was over 97% for all experiments (Table 1).

This behavior is in accordance with the work of Pathak et al. [38], who compared two methods of sample preparation. They decided to use vibrational polishing in favor of electropolishing in order to suppress pop-ins. In our work this is not necessary, because pop-ins affect indentation curves at a low depth below 200 nm only. Here we use pop-in effect as an indicator for the sample preparation quality.

**Figure 2:**
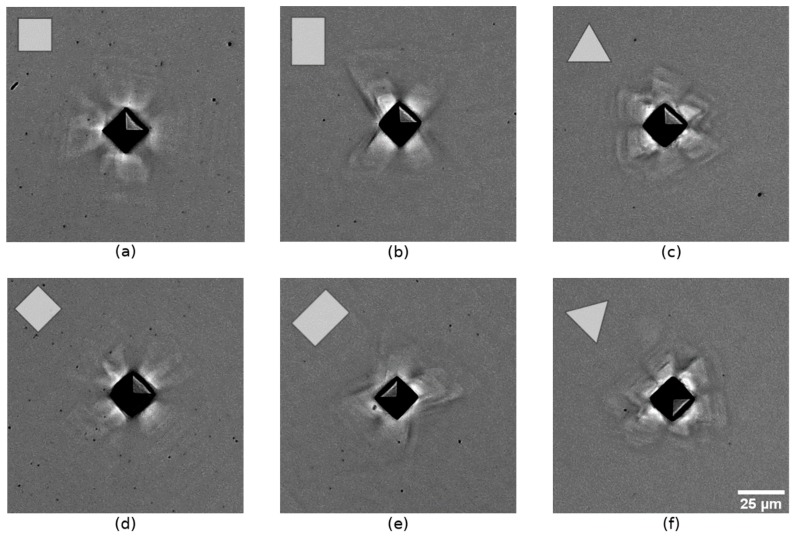
Optical images of pile-up patterns ($F_{max}$ = 60 mN) on three crystallographic orientations of aluminum single crystals (aligned patterns, (**a**): (100), (**b**): (110), (**c**): (111)). Indents on the same surfaces azimuthally rotated by 45° (misaligned patterns, (**d**): (100), (**e**): (110), (**f**): (111)).

The surface morphology around the indents was analyzed by optical microscopy. The original indents made with $F_{max}$ = 10 mN ($h_{max}$ ≈ 1.2 µm) are too small to be captured optically. To overcome this problem, indents made with a higher $F_{max}$ of 60 mN were analyzed (Figure 2). This approach is appropriate, since no significant difference of 60 mN and 10 mN indents is expected. This assumption is based on the work of Kucharsky and Jarzabek [39], who demonstrated that pile-up patterns with loads higher than 2 mN mainly resemble the crystal symmetry (Berkovich indentation of copper single crystals). The plastic surface deformation on (100)-, (110)-, and (111)-oriented samples show distinct similarity to the four-, two-, and three-fold symmetry. The images of misaligned indenter–crystal configuration (Figure 2d–f)) exhibit surface deformation patterns that are almost identical to the aligned indents.

### 4.2. Pile-Up and Sink-In Corrections

**Figure 3:**
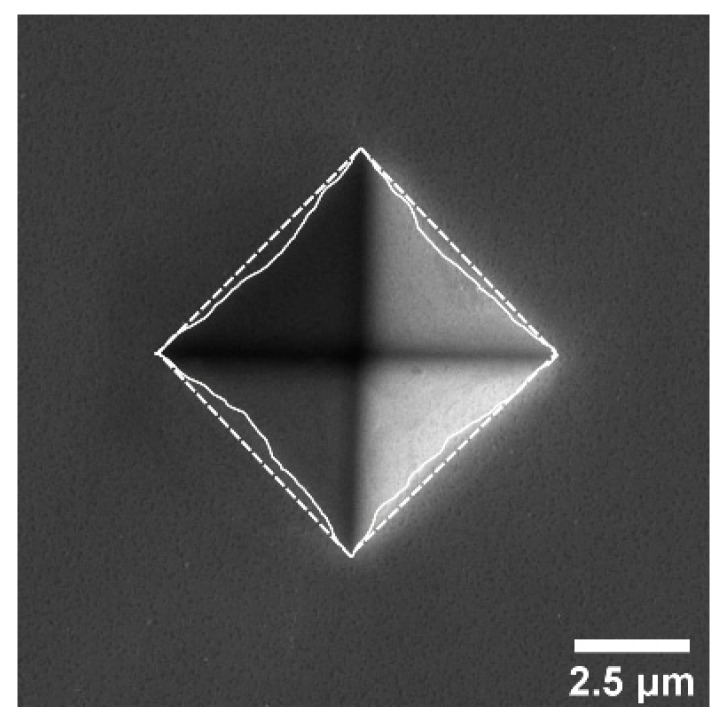
Scanning electron microscope (SEM) image of the indented area ($F_{max}$ = 10 mN) on the crystallographic (100) orientation of aluminum single crystal (aligned). $A_{p}$ is enclosed by the solid and $A_{c}$ by the dashed line.

**Figure 4:**
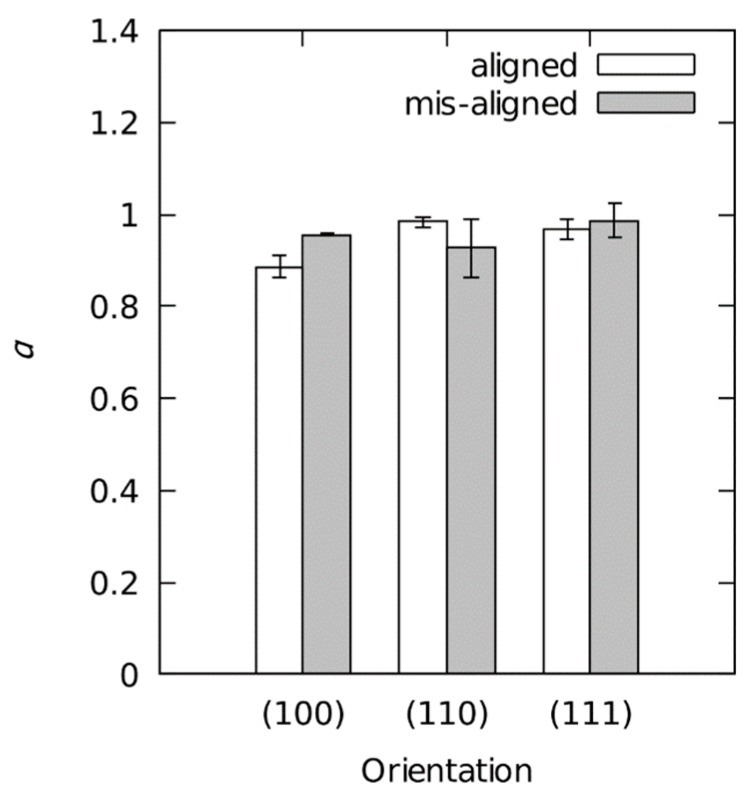
Dependence of the factor α on the crystal and indenter orientation. Average values with the error bar indicating the respective range ($\alpha_{max}-\alpha_{min}$).

Figure 3 depicts a (100)-oriented sample. In it, $A_{p}$ (solid line) is smaller than $A_{c}$ (dashed line) which leads to an α-factor less than 1. The overall results for the α-factor (Figure 4) confirm this trend ($A_{p}<A_{c}$) for all indenter–sample configurations. Furthermore, α seems to be dependent on the indenter orientation for the crystal orientation (100) and (110). No such dependency is observed for the (111) orientation.

The values of $h_{p}$ and $H_{t}$, calculated for one representative indentation curve ((110) orientation), are summarized in Table 2. Apparently, $h_{p}$ shows a value higher than the cross sectional $h_{c}$ determined by the Oliver and Pharr method. The consequence of the different contact depth is that the determined $H_{t}$ is lower than $H_{IT}$.

### 4.3. Influence of the Crystal Orientation on $H_{IT}$ and M

**Figure 5:**
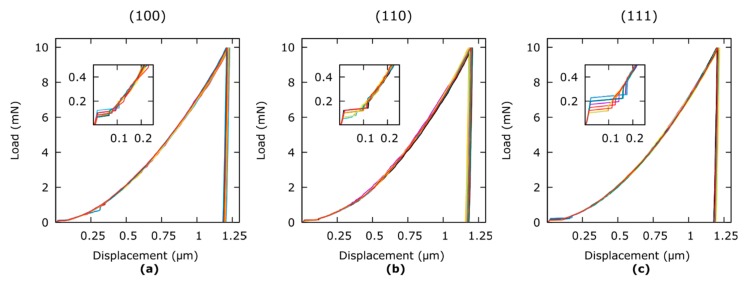
Indentation curves produced with the 6 × 6 arrays on differently oriented Al single crystal surfaces: (**a**) (100) orientation, (**b**) (110) orientation, and (**c**) (111) orientation.

The indentation curves on the basis of which the statistical data has been achieved are shown in Figure 5. The curves show good reproducibility, no visible influence from vibrations, and a distinguishable pop-in effect in the low-load region.

**Figure 6:**
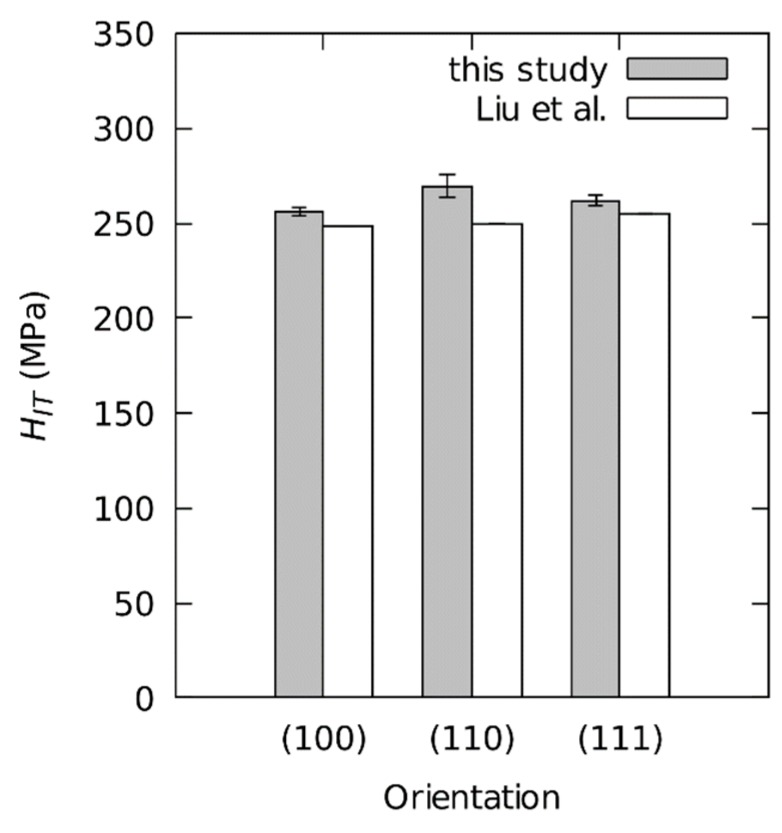
$H_{IT}$ for three crystallographic orientations of the aluminum single crystal (grey: this study, white: Liu et al. [8]).

Average $H_{IT}$ values for particular crystallographic orientations are demonstrated in Figure 6. Indentations on the (100)-, (110)-, and (111)-oriented samples result in the hardness values ($H_{IT}\pm \sigma_{H_{IT}}$) of 256.0 ± 2.2 MPa, 269.6 ± 5.9 MPa, and 261.6 ± 2.7 MPa respectively. The (110)-oriented sample has the highest hardness but also the highest standard deviation. The average hardness over all crystallographic orientations is 262.4 MPa with a range $R_{H_{IT}}=H_{IT,max}-H_{IT,min}$ = 13.6 MPa (5.1%). According to the range rule of thumb mentioned by Mandel [40] (R≈4σ), the standard deviation equals approximately 1.3%. In this case it originates exclusively from the differences in crystallographic orientation.

**Figure 7:**
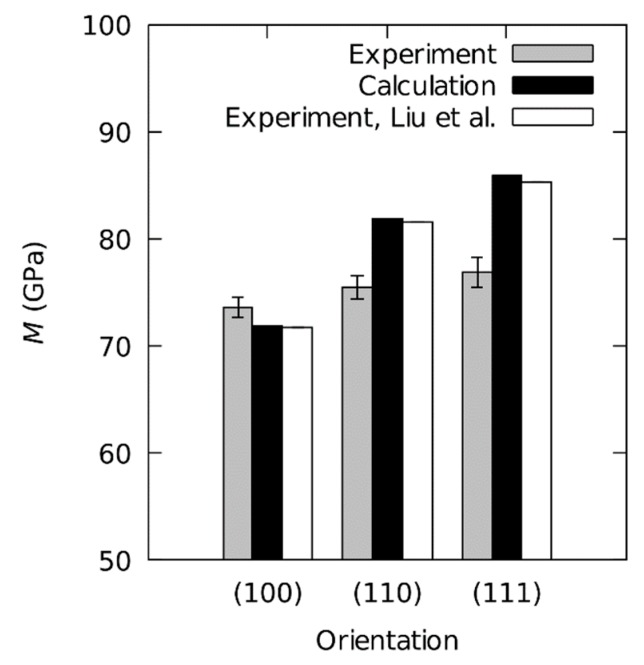
M for three crystallographic orientations of the aluminum single crystal. Grey shows this study (Experimental M); black shows calculated M according to Equation (1); white shows Liu et al. [8] (Experimental M).

Average values of M with the corresponding standard deviations are shown in Figure 7. The (100), (110), and (111) orientations result in the indentation modulus values ($M\pm \sigma_{M}$) of 73.6 ± 0.9 GPa, 75.5 ± 1.1 GPa, and 76.9 ± 1.4 GPa respectively. The standard deviation on all three samples is reproducible. The absolute values differ from the theoretical values for $E_{uvw}/\left(1-\nu^{2}\right)$ (79.1 GPa, 82.5 GPa and 86 GPa respectively, see Table 4). However, they show the same trend with the (110) orientation being elastically the softest and (111) the stiffest. Analogous to $H_{IT}$, the average indentation modulus over all orientations is 74.5 GPa with a range $R_{M}$ = 3.3 GPa (4.4%), and the standard deviation derived from crystallographic orientations is approximately 1.1%.

### 4.4. Influence of the Indenter Orientation on $H_{IT}$ and M

**Figure 8:**
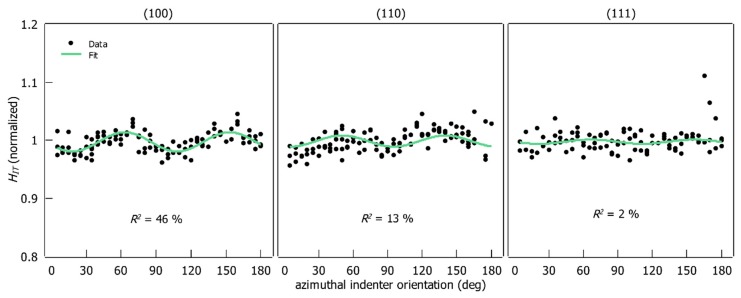
$H_{IT}$ (normalized to the mean values) as a function of the azimuthal indenter orientation on three crystallographic orientations.

**Figure 9:**
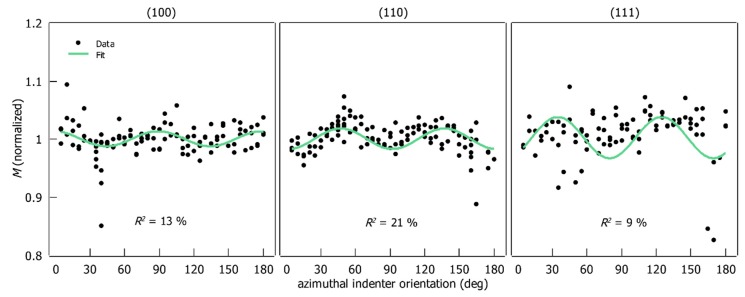
M (normalized to the mean values) as a function of the azimuthal indenter orientation on three crystallographic orientations.

$H_{IT}$ and M are plotted as a function of the azimuthal indenter orientation in Figure 8 and Figure 9 respectively. The absolute values are normalized with respect to the average of the corresponding data-set for better comparison. The fitted harmonic function from Equation (10) is shown as a green line along with the fit parameter $R^{2}$.

It appears that $H_{IT}$ and M vary periodically with the azimuthal indenter orientation for (100)- and (110)-oriented samples. Two periods for the investigated rotation range can be distinguished for these orientations. In contrast, no periodicity is observed for the (111)-oriented sample.

Each data set has outliers. In the preliminary test, an attempt was made to remove the outliers manually, but there was no significant improvement in the resulting fits. Therefore, the fits were performed on the raw data sets. The function from Equation (10) could be fitted successfully to the experimental data from (100)- and (110)-oriented samples. The $R^{2}$ of the fits lies between 12% and 46%. No satisfactory fit result for the (111) orientation could be achieved ($R^{2}<10$). Additionally, residual analysis was performed (see supplementary material, Figure S1). The error is evenly distributed along the x-axis (orientation) for $H_{IT}$ and M on the (100) orientation, with no systematic effects. For the (110) orientation the spread increases slightly from 150° to 185° ($E_{IT}$ and M). Since the fits for the (111) orientation do not correlate with the data, a residual analysis does not make sense here.

The period of the fitted function is 90° for all sample orientations, which conforms to the geometry of the indenter (quadratic pyramid). Additionally, σ and the fitted $x_{0}$ values for the (100) and (110) orientations are shown in Table 3. The fitted $x_{0}$ is partially higher than half of the corresponding σ.

## 5. Discussion

### 5.1. Quality of Sample Preparation

According to the analysis of the pop-in behavior (pronounced pop-in effect is on almost all load–depth curves), the applied sample preparation technique is ideal. Electropolishing affects the first 100–200 nm of the indentation curves by introducing pop-ins. Yet, $H_{IT}$ and M are calculated at depths of greater than 1100 nm (at maximum load and at the unload-part of the indentation curve respectively). Thus, the influence of pop-ins on the indentation results is negligible. Furthermore, the pop-in effect is utilized to qualify mechanical influence through sample preparation. Since a pop-in indicates a dislocation-free region of a single crystal, it can be concluded that the sample is not strain-hardened (97% of the curves show pop-ins). Therefore, the influence of the applied sample preparation on the *derived material properties* of aluminum is very low. The possible influence from the surface oxides and the remains of the polishing agents is not part of these studies.

Characteristic surface deformation patterns on all three crystallographic orientations correspond to the correct crystallographic planes almost independently from the azimuthal indenter orientation. Therefore, the correct sample orientation and the integrity of the crystal lattice can be confirmed on the basis of the pile-up patterns. The plastic deformation seems to depend mainly on the crystallographic orientation and not on the indenter orientation for the chosen indentation depth which complies with the findings of Wang et al. [18] and Kucharski and Jarzabek [39].

### 5.2. Correction for Plastic Behavior

According to the presented SEM image of the indent (Figure 3) and the determined α-values (Figure 4), the sink-ins are evident. The magnitude of the sink-in appears to depend on the azimuthal indenter orientation. Nevertheless, the $h_{p}$ and $H_{t}$ values clearly indicate a pile-up, not a sink-in (determined on the basis of the plastic and elastic indentation work relationships of Tuck et al. [35]). Since the true contact area can be reliably seen from the SEM images, the calculation of $H_{t}$ must be discarded for the case of aluminum single crystals. Since α shows a complicated behavior depending on both the crystallographic orientation of the sample and the azimuthal orientation of the indenter, the $H_{IT}$ and M results cannot be corrected with a single factor. To determine the α-factor SEM-images of all indents have to be captured and evaluated according to the proposal of McElhaney et al. [34]. This is not necessary in our work, because α deviates only slightly from 1. Therefore, the pile-up/sink-in correction can be neglected. Nevertheless, it should be kept in mind that the determined $H_{IT}$ and M values are underestimated because of the observed sink-in effect.

### 5.3. Influence of the Crystal Orientation on $H_{IT}$ and M Values

The achieved hardness data are in good agreement with the results reported by Liu et al. in 2015 [8] and are lower than the results reported by Liu in 2014 [41]. However, the surface quality was not analyzed through pop-in analysis and no information about data scatter is provided in these references. It is assumed that the hardness difference in the literature originates in different sample preparation.

The indentation direction on the (111)-oriented samples is perpendicular to the slip direction of the fcc slip system, as mentioned by Rösler et al. [42] (slip plane of the type {111}, slip direction of type $\langle 1\bar{1}0\rangle$). Therefore, this sample orientation should exhibit the greatest hardness. In our result, the (110)-oriented sample shows the greatest $H_{IT}$ value. The reason for this is the high data scatter. Therefore, the hardness anisotropy matches the theory. Overall, the hardness values are probably close to the intrinsic material properties. In addition, the standard deviation achieved in this study is very low.

To evaluate the magnitude of the averaging effect and crystal anisotropy the experimental data is compared with the calculated directional Young’s moduli (according to Equation (1)), indentation moduli (according to Equations (2) and (6)), and two literature sources (see Table 4). Additionally, a ratio of the stiffest to the softest direction $M_{111}/M_{100}$ has been introduced to compare the derived anisotropy of the different elastic moduli.

The absolute indentation moduli are slightly lower than predicted. This can be attributed to machine compliance. Furthermore, M is underestimated because of the sink-in effect. Nevertheless, the resulting experimental $M_{111}/M_{100}$ ratio shows a very good agreement with the theory. It is also only slightly higher than the ratio approximated indentation moduli.

A significant difference to the $M_{111}/M_{100}$ ratio presented by Liu et al. [8,41] must be noted. Their result almost completely coincides with the value of the directional Young’s modulus. However, this does not consider the averaging effect (either experimentally or by FEM). According to this effect the $M_{111}/M_{100}$ should be lower than $E_{111}/E_{100}$ ratio (as discussed in Section 2.2). This behavior is clearly observable from the results achieved in the present study. Here, $M_{111}/M_{100}$ is significantly lower than $E_{111}/E_{100}$, which demonstrates the indentation averaging effect. Furthermore, the achieved data is in very good agreement with the experimental and theoretical results of Vlassak and Nix [16].

The information about how the elastic anisotropy is altered by the averaging effect is very important because it describes the real-life behavior of the material. These results are essential for a better understanding of material behavior and can be implemented in the indentation data spread analysis of polycrystalline material.

### 5.4. Influence of the Azimuthal Indenter Orientation 

The influence of the indenter orientation on the indentation modulus has been calculated by Vlassak and Nix [16] for cubic single crystals indented with a flat triangular punch indenter (three-fold symmetry). However, no *experimental studies* on the influence of the azimuthal indenter orientation on mechanical properties of single crystal aluminum have been found.

The chosen sine function has proven to be suitable for describing the influence of the azimuthal indenter orientation on hardness for the (100)- and (110)-oriented specimens. A great fraction of the data scatter could be attributed to the influence of the azimuthal indenter orientation with the fitted function.

It is experimentally demonstrated that the indenter orientation has a measurable effect on $H_{IT}$ and M (Figure 8 and Figure 9). This is proven by the fact that the period of the observed effect matches the indenter geometry. Additionally, the periodic modulation of hardness and indentation modulus only occurs on (100)- and (110)-oriented specimens whose symmetry (four-fold and two-fold) is related to the quadratic pyramid of the Vickers indenter (four-fold). Therefore, the periodic behavior of the measured parameters is attributed to the combined effect of the indenter geometry and the symmetry type of the indented surface. 

These findings extend the results of Wang et al. [18] on copper single crystals to aluminum and quantify the effect on $H_{IT}$ and M. The further analysis of the slip systems and three-dimensional anisotropy of the elastic modulus, which are the physical basis for this effect, is beyond the scope of this study.

Future work should aim to transfer the single influences elaborated in this study to polycrystalline aluminum. To accomplish this, pure polycrystalline aluminum will need to be examined by grid nanoindentation. The variation of hardness and indentation modulus on polycrystalline aluminum is then to be compared with the integrated influences determined in this study. Also, the influence of dislocation density on plastic behavior could be considered thoroughly in further work.

## 6. Conclusions

- Distinctive surface deformation patterns (crystal integrity) and the pop-in effect (low dislocation density) demonstrated that the combination of mechanical and electrolytic sample preparation has almost no influence on the acquired results.
- No correction method for the true contact area could be applied due to the directionality of the sink-in behavior.
- The range of the effect of crystallographic orientation on $H_{IT}$ was found to be 7.3% relative to average hardness, which corresponds to a σ of 1.8%.
- The variation of indentation modulus on three different crystallographic orientations can be assigned to elastic anisotropy of aluminum. The anisotropy of the indentation modulus is reduced as a result of indentation averaging. This is correctly predicted by the implemented data analysis model.
- The periodic behavior of $H_{IT}$ and M as a function of azimuthal indenter orientation is assigned to the combined effect of indenter geometry and crystal symmetry. Therefore, only the interaction of even-numbered symmetries results in periodic behavior.
- Through the analysis of a harmonic function fitted to the experimental data, a significant fraction of the data scatter can be assigned to the effect of the azimuthal orientation of the indenter. For the first time, it is possible to quantify this effect on the basis of experimental data.

## Figures and Tables

**Table 1:** Proportion of the load–depth curves that exhibit pop-ins. The analysis is based on the curves from experiments on the influence of the crystal orientation and the azimuthal indenter orientation.

Sample Orientation	(100)	(110)	(111)
Influence of crystal orientation	100%	98%	97%
Influence of azimuthal indenter orientation	97%	100%	100%

**Table 2:** Determined $h_{p}$ and $H_{t}$ on the basis of one indentation curve ($F_{max}$ = 10 mN, (110) direction) in comparison to $h_{c}$ and $H_{IT}$ determined according to the method of Oliver and Pharr.

$h_{c}$	1.24 µm
$h_{p}$	1.28 µm
$H_{IT}$	249.5 MPa
$H_{t}$	235.2 MPa

**Table 3:** Fitted values for $x_{0}$ in comparison to σ of the corresponding data sets from the measurement series of the azimuthal indenter orientation influence on $H_{IT}$ and M.

Sample Orientation	(100)	(110)
$\sigma_{H_{IT}}$	1.7%	2.0%
$x_{0,H_{IT}}$	1.6%	1.0%
$\sigma_{M}$	2.6%	2.6%
$x_{0,M}$	1.3%	1.8%

**Table 4:** Comparison of $E_{IT}$ values from literature and obtained in this study from the crystal orientation analysis. The values are given with the precision of the corresponding original publication.

	$M_{uvw}$ (GPa)				
Calculation Method	(100)	(110)	(111)	$M_{111}/M_{100}$	Ref.
$E_{\left[uvw\right]}/\left(1-\nu^{2}\right)$, Equation (1)	72.6	82.5	86.5	1.191 ($E_{111}/E_{100}$)	this study
$M_{\left[uvw\right]}$, Equations (2)–(6)	79.1	82.5	86.5	1.019	
M, Equation (8)	73.6 ± 0.9	75.5 ± 1.1	77.0 ± 1.4	1.046	
M, Equation (8)	71.8	81.6	85.4	1.189	[8,41]
M, Equation (8)	77		79	1.015	[16]
numeric approximation	79	80	81	1.029	[16]

## Supplementary Materials

The following are available online at https://www.mdpi.com/1996-1944/12/22/3688/s1, Figure S1: Residual analysis of the fit to the azimuthal indenter orientation.
