# Supplementary material for: Nanoindentation of Aluminum Single Crystals: Experimental Study on Influencing Factors
Source: Materials (Basel). 2019 Nov 8;12(22):3688. doi: 10.3390/ma12223688 (PMC6887952; doi:10.3390/ma12223688)
Supplement: Supplementary file 1 [file materials-12-03688-s001.pdf]

materials

MDPI

Article

Nanoindentation of Aluminum Single Crystals: Experimental Study on Influencing Factors

Pavel Filippov [1]* and Ursula Koch [2]

[1] Munich University of Applied Sciences, Munich, Germany; Department of Earth- and Environmental Science, Ludwig-Maximilian-University, Munich, Germany; pavel.filippov@hm.edu

[2] Munich University of Applied Sciences, Munich, Germany; u.koch@hm.edu

* Correspondence: pavel.filippov@hm.edu; Tel.: +49(0)89-1265-3649

Received: 10 October 2019; Accepted: 6 November 2019; Published: 8 November 2019

Keywords: Nanoindentation; aluminum; single crystal, micromechanics

1. Supplementary material: Residual analysis

The residuals e are calculated as the difference of the measured value y and the modelled value y':

$$e = y - y' \quad (1)$$

The respective residuals are calculated on the basis of data shown in Figure 8 (H_{IT}) and Figure 9 (M).

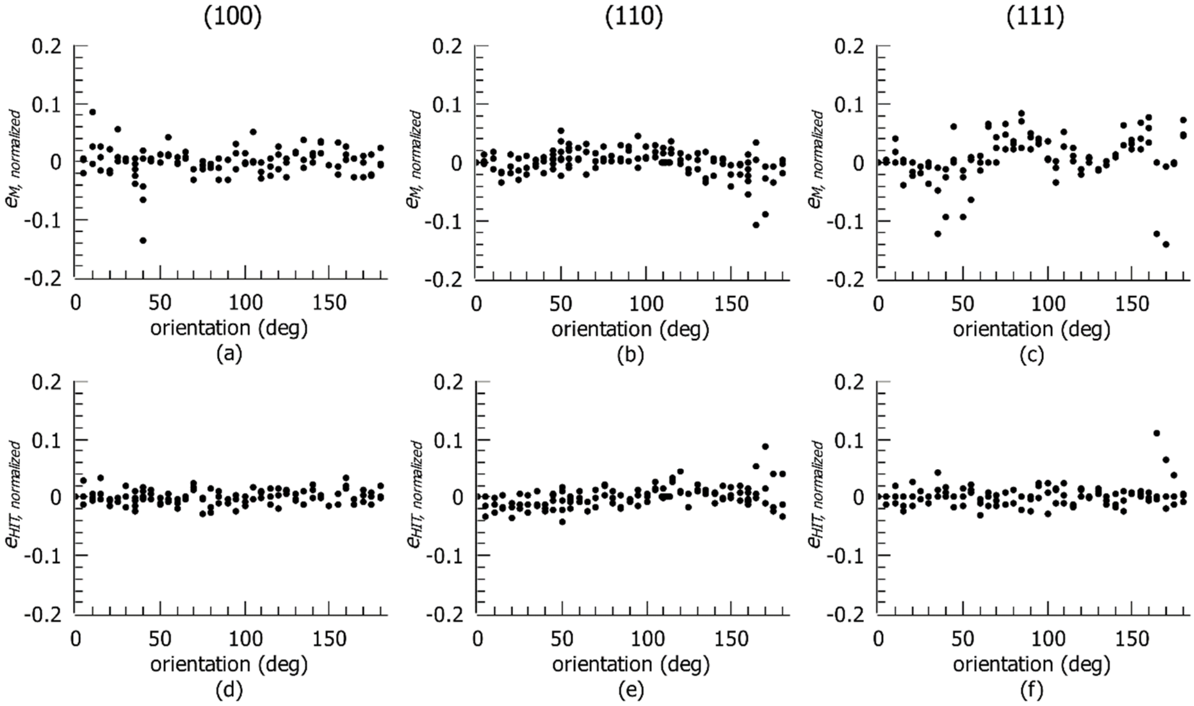

Figure S1 – Residual analysis of the fit to the azimuthal indenter orientation: $e_{M,normalized}$: a) (100)-orientation, (b) (110)-orientation and (c) (111)-orientation. $e_{HIT,normalized}$: a) (100)-orientation, (b) (110)-orientation and (c) (111)-orientation

© 2019 by the authors. Submitted for possible open access publication under the terms and conditions of the Creative Commons Attribution (CC BY) license (http://creativecommons.org/licenses/by/4.0/).
